# Posterior Reversible Encephalopathy Syndrome Associated with FOLFOX Chemotherapy

**DOI:** 10.1155/2013/306983

**Published:** 2013-02-27

**Authors:** Luiz Carlos Porcello Marrone, Bianca Fontana Marrone, Tharick Ali Pascoal, Lucas Porcello Schilling, Ricardo Bernardi Soder, Sheila Schuch Ferreira, Giovani Gadonski, Jaderson Costa da Costa

**Affiliations:** Hospital São Lucas/Instituto do Cérebro (Inscer), Pontificia Universidade Católica do Rio Grande do Sul (PUCRS), Avenida Ipiranga 6690, 90610-000 Porto Alegre, RS, Brazil

## Abstract

Posterior reversible encephalopathy syndrome (PRES) is a clinicoradiologic entity characterized by headaches, altered mental status, seizures, visual loss, and characteristic imaging pattern in brain MRI. The cause of PRES is not yet understood. We report a case of a 27-year-old woman that developed PRES after the use of FOLFOX 5 (oxaliplatin/5-Fluoracil/Leucovorin) chemotherapy for a colorectal cancer.

## 1. Introduction

Posterior reversible encephalopathy syndrome (PRES) is a clinicoradiologic entity characterized by headaches, altered mental status, seizures, and visual loss and is associated with white matter vasogenic edema predominantly affecting the occipital and parietal lobes of the brain [1]. The cause of PRES is not yet understood.

Autoregulatory failure with resultant vasodilation, as seen in hypertensive encephalopathy, is often cited as the underlying mechanism. On the other hand, vasospasm with ischaemic change is also observed in some patients [2, 3].

The most characteristic imaging pattern in PRES is the presence of edema involving the white matter of the posterior portions of both cerebral hemispheres, especially the parieto-occipital regions, in a relatively symmetric pattern that spares the calcarine and paramedian parts of the occipital lobes [1]. However, other structures (such as the brain stem, cerebellum, and frontal and temporal lobes) may also be involved, and although the abnormality primarily affects the subcortical white matter, the cortex and the basal ganglia may also be involved [4].

Numerous factors can trigger the syndrome, most commonly: acute elevation of blood pressure, abnormal renal function, and immunosuppressive therapy. Other possible etiologies are eclampsia, transplantation, neoplasia and chemotherapy treatment, systemic infections, and a renal disease, acute or chronic [1, 5].

## 2. Case Report

A 27-year-old woman with an advanced colorectal adenocarcinoma with peritoneal metastasis (T3N1 M1; clinical stage IV) was admitted in the emergency room due to abdominal pain, vomiting, and suspect of intestinal obstruction. She was submitted to resection of the primary tumor 3 months before the admission and she underwent the second cycle of chemotherapy with FOLFOX 5 (oxaliplatin/5-fluorouacil/leucovorin).

She was without fever and her blood pressure was 200/120 mmHg. In the basic blood test, there were no abnormalities except an increased of creatinine (1.9 mg/dL, previous creatinine was 0.7 mg/dL three months ago).

During the hospital stay she presented two seizures and after the second seizure she developed confusional mental state that progressed to obnubilation. In her clinical examination, there was no focal neurologic deficit; however the patient was poorly cooperative secondary to her confusional state. The patient was submitted to a gradual reduction of the blood pressure and it was initiated with anticonvulsive drugs (valproate sodium 1000 mg/day).

**Figure 1:**
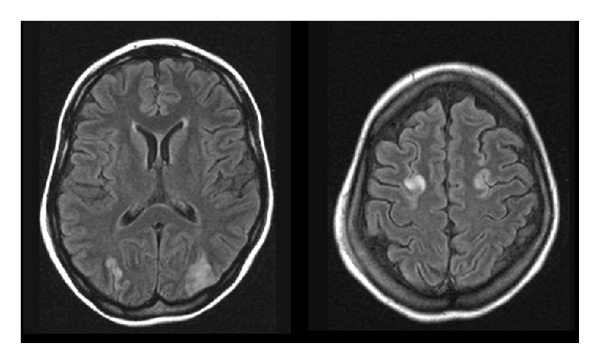
Brain MRI-FLAIR showing an increase of signal in both occipital and frontal lobes.

A brain MRI (FLAIR/T2) was performed two days after the onset seizure. This first brain MRI showed an increase of signal in both occipital and frontal lobes (in watershed zones), with a symmetric pattern (Figure 1). After three days, when the patient became more awake, she reported a bilateral visual disturbance that persisted for one week.

After ten days, a new brain MRI was performed that shows no evidence of edema or other abnormalities. Three months after the situation described, the patient died due to complications of cancer.

## 3. Discussion

This syndrome was firstly described by Hinchey and colleagues as a clinicoradiologic entity with numerous triggers. In 2000, Casey et al. established the term posterior reversible encephalopathy syndrome (PRES). The pathophisiology is usually associated with cerebral blood flow autorregulation disruption and/or endothelial dysfunction. Cytotoxic medications can cause endothelial dysfunction. Hypertension is the main cause of PRES. However, PRES is not associated with hypertension in 20 to 30% of patients, and a lack of hypertension does not rule out the possibility of PRES [1, 6].

Previous reports described the association between chemotherapy and antiangiogenic drugs with PRES. No single antineoplastic class or agent has been consistently associated with PRES although some chemotherapeutic agents may cause direct CNS microvascular injury. There are few articles in the literature that report the correlation between PRES, cancert and neoplastic treatments (like chemotherapy). 

FOLFOX (oxaliplatin/5-fluorouacil/leucovorin) was approved by the US Food and Drug Administration as indicated for first-line therapy for advanced colorectal cancer in 2000. One of the most common adverse effects of the FOLFOX is the neuropathy [7].

The relationship between PRES and FOLFOX was not well established. In a review performed in Pubmed, we found two cases that showed the use of FOLFOX preceding this syndrome [8, 9].

In 2007, Skelton and colleagues reported a case of PRES in a 19-year-old woman with metastatic adenocarcinoma of the rectum who received modified FOLFOX6 chemotherapy. She presented seizures and altered mental status ten days after the start of fourth cycle of chemotherapy. Her blood pressure was increased at 170/110 mmHg with and increased creatinine (1.5 mg/dL). Brain MRI demonstrated a hyperintensity in the white matter of the posterior hemispheres. One week after the hospitalization, a follow-up MRI on the brain revealed a dramatic improvement in the MRI image abnormalities, and this was consistent with a resolving picture of PRES [8].

In 2011, Kim et al. reported a case of a 52-year-old woman with advanced gastric cancer, presented with low-back pain due to spinal metastasis at the 4th lumbar vertebra. The primary tumor was not resectable, and 10 cycles of chemotherapy with FOLFOX had been completed. An elective surgical spinal decompression and stabilization was scheduled. Twenty-three days after the end of chemotherapy, a generalized tonic-clonic seizure occurred. Her systolic blood pressure was 166 mmHg at the time of the seizure and no electrolyte abnormality was observed. After the seizure, a brain computed tomography was conducted, with no definite abnormality. One day after the seizure a brain MRI was conducted, and a signal change was observed in both parieto-occipital lobes in T2-weighted and FLAIR images. As a result of typical imaging features, PRES was diagnosed. An antiepileptic drug was administered and no more seizures were reported [9].

In this paper, we showed a new case of PRES after the use of FOLFOX with imaging findings not yet described in this situation. In this case, hypertension and/or renal failure can be another trigger for this syndrome. The first brain MRI of our patient showed typical increased signal intensity in both occipital lobes but also revealed a watershed/junctional pattern of distribution affecting the superior frontal sulcus of frontal lobes.

In typical PRES, the parietal and occipital lobes are most commonly affected, followed by the frontal lobes. However, in watershed imaging distribution of PRES, three hemispheric pattern variants involving cortex, subcortical, and deep white matter to varying degrees may be encountered with similar frequency: holo-hemispheric (23%), superior frontal sulcus (27%), and primary parietal-occipital (22%) [10]. These demarcate border zones between anterior, middle, and posterior circulations and reflect the junctional/watershed nature of PRES. There is a fourth imaging pattern distribution named “partial or asymmetric expression of the primary patterns” (28%), which occurs when the previous three forms are found in a partial way or asymmetric fashion. Here, we describe the first reported case of PRES with a watershed pattern affecting both superior frontal sulcui after the use of FOLFOX.

PRES caused by cytotoxic medications should be managed with control of hypertension, management of seizures, and withdrawal medication. The initial aim of treatment in hypertension is to lower the diastolic pressure to about 100 mmHg, with the maximum initial fall in BP not exceeding 25 percent of the presenting value. More aggressive lowering may reduce the blood pressure below the autoregulatory range, possibly leading to ischemic events. Oral antihypertensive agents are not usually effective in lowering the blood pressure to an appropriate range in hypertensive crises to prevent and treat PRES.

Patients should also receive antiepileptic medications that can probably be safely tapered as symptoms and neuroimaging findings resolve, usually after one to two weeks. In some cases patients have reported seizures months after PRES, and these patients were maintained on antiepileptic drug therapy.

Removal of the cytotoxic drug is usually recommended in cases of PRES associated with cytotoxic agents. It is not recommended that agents known to induce PRES be reintroduced, as recurrence has been reported in this setting.

In summary, PRES is an entity not well known by neurologists and oncologists and the delay in diagnosis can lead to a worse clinical recovery. Due to the increase in the number of neuroimaging studies that have been conducted as well as the increase of cases of patients submitted to chemotherapies treatments that can induce PRES, this syndrome is becoming more frequent. A peculiar neuroimaging pattern observed by T2/FLAIR signal alteration is first reported in FOLFOX chemotherapy.
